# Hyperthyroidism, hypothyroidism, thyroid stimulating hormone, and dementia risk: results from the NHANES 2011–2012 and Mendelian randomization analysis

**DOI:** 10.3389/fnagi.2024.1456525

**Published:** 2024-10-23

**Authors:** Xixi Sheng, Jixiang Gao, Kunfei Chen, Xuzhen Zhu, Yu Wang

**Affiliations:** ^1^Department of Neurology, Hangzhou TCM Hospital Affiliated to Zhejiang Chinese Medical University, Hangzhou Hospital of Traditional Chinese Medicine, Hangzhou, China; ^2^Department of Acupuncture Rehabilitation, Hangzhou TCM Hospital Affiliated to Zhejiang Chinese Medical University, Hangzhou Hospital of Traditional Chinese Medicine, Hangzhou, China

**Keywords:** dementia, hyperthyroidism, hypothyroidism, thyroid stimulating hormone, NHANES, Mendelian randomization

## Abstract

**Introduction:**

As the world ages, dementia places a heavy burden on society and the economy, but current methods of diagnosing dementia are still limited and there are no better therapies that target the causes of dementia. The purpose of this work is to explore the relationship between thyroid disease, thyroid stimulating hormone (TSH) concentrations, free tetraiodothyronine (FT4) concentrations and cognitive function.

**Methods:**

This study utilized cognitive function and thyroid data from the 2011–2012 National Health and Nutrition Examination Survey (NHANES) to assess the relationship between different groups of TSH and FT4 concentrations and cognitive function using weighted logistic regression and restricted cubic spline (RCS), and then used two-sample Mendelian Randomization (MR) to assess the causal relationship between hyperthyroidism, hypothyroidism, TSH and FT4 concentrations with dementia.

**Results:**

Our analysis of the 2011–2012 NHANES data showed that the individuals with low TSH concentrations had higher Alzheimer’s Disease Word List Registry Consortium1 (CERAD1) and CERAD.delay.recall scores than individuals with high TSH concentrations, and individuals with low FT4 concentrations had higher CERAD3 and Animal Fluency Test scores than individuals with high FT4 concentrations. Our results also showed a non-linear relationship between serum TSH and FT4 concentrations and the Animal Fluency Test. The TSH concentrations within the range of 1.703 to 3.145 mIU/L exhibit a positive correlation with Animal Fluency Test, whereas concentrations outside this range are negatively correlated with Animal Fluency Test. The FT4 concentrations exhibited a positive correlation with Animal Fluency Test to the left of the FT4 concentrations inflection point (0.849 ng/L), whereas to the right of this inflection point, correlation was negative. MR analysis results further indicate that genetic predisposition to hyperthyroidism may be associated with a reduced risk of dementia and vascular dementia(VaD). Conversely, genetic predisposition to hypothyroidism appears to be linked with an increased risk of dementia and VaD. Additionally, genetic predisposition to elevated TSH concentrations may be correlated with a heightened risk of risk of Alzheimer’s disease (AD).

**Conclusion:**

This study provides evidence of a nonlinear relationship between TSH and FT4 concentrations and cognitive function, with hyperthyroidism decreasing the risk of dementia and VaD, hypothyroidism increasing the risk of dementia and VaD, and elevated serum TSH concentrations increasing the risk of AD. Furthermore, prioritizing early detection, diagnosis, and treatment through the assessment of thyroid function in individuals at high risk for developing dementia is of paramount importance. This strategy has the potential to significantly contribute to the prevention and deceleration of dementia progression.

## 1 Introduction

Dementia is a syndrome of acquired cognitive impairment that significantly impairs patients’ ability to live, learn, work, and communicate and is a leading cause of death and disability among older adults (Lin et al., 2015). The most common type of dementia is Alzheimer’s disease(AD), which accounts for about 60–70% of cases. In contrast, others include vascular dementia(VaD), frontotemporal lobe dementia(FTD), dementia with Lewy bodies(DLB), or a combination of several types (called mixed dementia) (WHO, 2023). The prevalence of dementia is estimated to be 4.3%–6.4% in people over 60 years of age and 22.1%–30.1% in people over 85 years of age (Ferri et al., 2005; Noguchi-Shinohara et al., 2013). Dementia cases are expected to reach 152.8 million by 2050 because the world’s population is aging (GBD 2019 Dementia Forecasting Collaborators, 2022). Dementia is the leading cause of functional disability, resulting in an enormous social and economic burden that is projected to cost $2.8 trillion globally by 2030 (Thornton et al., 2023). However, our understanding of the causes and progression of dementia remains limited, and there are no improved therapies that address the causes of dementia (Salehipour et al., 2022). Therefore, through risk factor management and appropriate treatment, it is possible to intervene and slow or stop the progression of dementia.

Thyroid status has emerged as a potential independent risk factor for reversible cognitive impairment over the past two decades (Yoshimasu et al., 1991; Ganguli et al., 1996). Thyroid dysfunction, i.e., hyperthyroidism and hypothyroidism, have different clinical features; hyperthyroidism is characterized by decreased levels of thyroid stimulating hormone (TSH) and increased levels of thyroid hormone (TH) and hypothyroidism is characterized by increased levels of TSH and decreased levels of TH. Both hyperthyroidism and hypothyroidism can be classified as clinical or subclinical and are characterized by elevated or suppressed TSH concentrations without clinical changes or abnormalities in TH concentrations (Ross et al., 2016). TSH regulates thyroid growth and TH production (Shahid et al., 2023). There are two active forms of TH, triiodothyronine (T3) and tetraiodothyronine or thyroxine (T4) (Raymaekers and Darras, 2017). T4 has a longer half-life than T3 and is considered the main circulating form of TH. Of total TH production, 93% is T4 and 7% is T3 (Bárez-López and Guadaño-Ferraz, 2017). Normal thyroid function is an important factor in the maintenance of a desirable cognitive state during aging.

TH has receptors in most tissues of the body, so they are highly influential in the myriad processes of metabolism and homeostasis in the body. They influence brain metabolism, neurogenesis, myelin formation, and cellular repair throughout the lifespan (Calzà et al., 2015). TH disorders can lead to a variety of developmental, metabolic, and age-related disorders and may be associated with dementia (Cappola et al., 2015) and cognitive impairment (Pasqualetti et al., 2015). A prospective longitudinal study evaluating whether abnormal TH levels predict dementia found that higher free T4 (FT4) levels predicted new-onset dementia in older men, independent of traditional risk factors for cognitive decline (Yeap et al., 2012). Higher TSH concentrations was associated with a diagnosis of dementia in a community-based study of 194 individuals (Ganguli et al., 1996). In contrast, a cross-sectional study found that patients with AD had significantly lower TSH concentrations than controls, independent of other risk factors, and lower TSH concentrations was associated with a more than twofold increased risk of AD (Van Laere et al., 2004).

Dementia and thyroid dysfunction are both common in the elderly. Although there have been previous studies examining the relationship between thyroid disease, TSH, FT4 and dementia, the results have been inconsistent, while most studies have examined the relationship between thyroid function and AD. The inconsistency of these findings may be attributable to the limited sample sizes of extant observational studies, insufficient adjustment for critical variables, and the absence of assessments for various types of dementia. Therefore, this study proposes to investigate the association between thyroid function and dementia using genetic Mendelian randomization (MR) analysis, a tool that uses genetic variants as an instrumental variable to investigate causal relationships between clinical traits and disease phenotypes (Lawlor et al., 2008). MR is superior to observational studies that control for confounders and reverse causation because genetic alleles are randomly assigned during meiotic subtraction and are not affected by environmental factors (Verduijn et al., 2010). The MR study has been described as a “naturally occurring, randomized, double-blind study” that is complementary to randomized controlled trials (RCTs).

Therefore, this study aims to explore the relationship between different types of dementia and thyroid dysfunction, TSH, and FT4 concentrations by analyzing data from the National Health and Nutrition Examination Survey (NHANES) 2011–2012 and to assess the causality of these associations with MR analysis.

## 2 Materials and methods

### 2.1 Research data from NHANES

**FIGURE 1:**
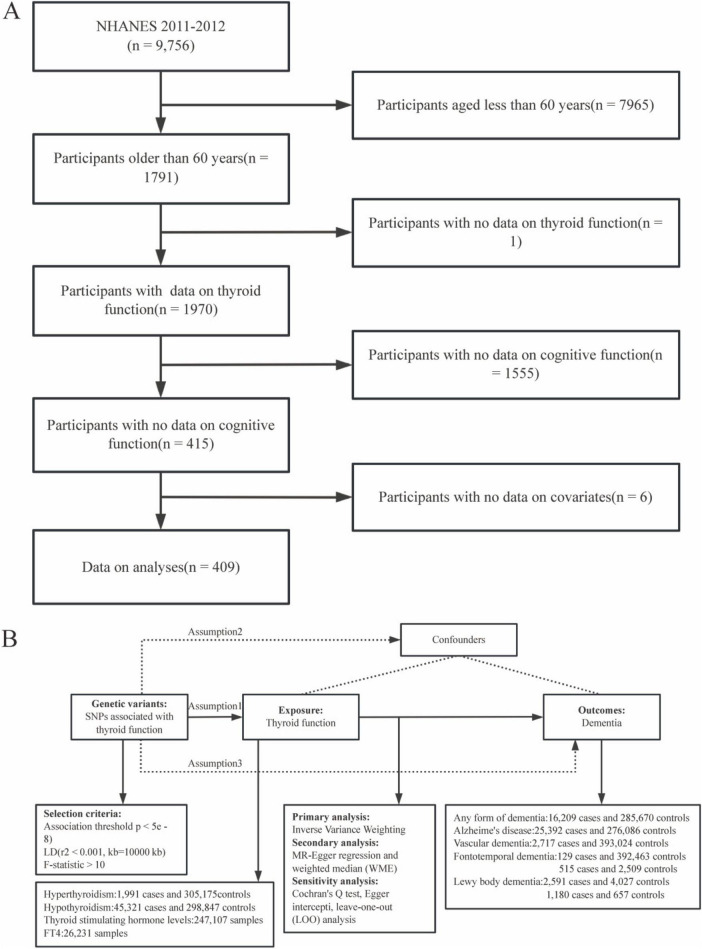
Research flowchart for this study. Research flowchart for this study. **(A)** Flowchart of the selection of eligible participants in the National Health and Nutrition Examination Survey (NHANES). **(B)** Flowchart of Mendelian Randomization(MR) and assumptions: Assumption 1: exposure is strongly associated with genetic variants; Assumption 2: confounders are not associated with genetic variants; Assumption 3: The effect of genetic variants on outcomes should be mediated only by the exposure of interest.

The NHANES is a cross-sectional survey designed to provide a representative sample of the United States (U.S.) civilian non-institutionalized population (CDC, 2016). In these analyses, we propose to study the relationship between thyroid function and cognitive function by selecting data from the NHANES database. Serum thyroid function TSH and FT4 have been measured in several NHANES cycles (1999–2002), but unfortunately, these data on cognitive function were incomplete and therefore cannot be used as additional measures of thyroid functional status. Cognitive function was also measured during several NHANES cycles (2013–2014), but unfortunately, data on thyroid function were not included in these data and therefore could not be used as an additional measure of cognitive function. Meanwhile, 2019–2020 NHANES data are available for thyroid function and cognitive function, but due to the coronavirus disease 2019 (COVID-19) pandemic, the NHANES program is suspending field operations in March 2020, and therefore any analyses based solely on the 2019-March 2020 data would not be generalizable to the U.S. civilian non-institutionalized population. Also, the cognitive function scores in the 2019–2020 NHANES data are partially different from the 2011–2012 cognitive function scores, and the two cycles cannot be fully merged. The NHANES data for the period 2021.8–2023.08 is also not available to the public at this time. Therefore, we conducted a cross-sectional analysis using data from one cycle (2011/2012) of the NHANES data with both thyroid function data and cognitive function scores, including 9756 subjects. Previous studies have found an independent association between subclinical hyperthyroidism and cognitive dysfunction in older adults (Ceresini et al., 2009) and the cognitive function scores in the NHANES database are only for older people over the age of 60. Therefore, the inclusion criteria for this study were primarily people over the age of 60. Descriptive data of various paper-based cognitive assessments of community-dwelling older adults stratified on the basis of age and education: The study categorized the older adults into three groups of 60–69, 70–79 and 80–98 years of age, and found that younger older adults and those with higher education scored higher overall and domain scores (Brigola et al., 2018). Therefore, this paper categorized the age into three groups of 60–69, 70–79, and 80+ to avoid false-negative results due to over-stratification by age. In addition, among all subjects over 60 years of age, we excluded individuals who (1) lacked an indicator of thyroid function testing. (2) Subjects without cognitive function scores. (3) Subjects missing the following data, including sex, age (chronological age), race/ethnicity, BMI, household income (poverty income ratio, PIR), education level, alcohol consumption, and smoking status. The final study sample size was 409 subjects. The specific process for this is shown in Figure 1A. Study protocols for NHANES were approved by the NCHS ethics review board. Signed informed consent was obtained from all adult participants.

### 2.2 Thyroid function summary

Venous blood samples for thyroid function were obtained in accordance with the standard protocol (NHANES (2011–2012) Procedure Manual). FT4 levels were measured by a two-step enzyme-linked immunosorbent assay. TSH levels were measured by the Access High Sensitivity TSH assay, a third-generation two-site immunoassay (Hollowell et al., 2002; Kim et al., 2022). Hyperthyroidism was diagnosed when TSH levels were below 0.45 mIU/L and FT4 levels were above 1.6 ng/dL. Hypothyroidism was diagnosed when TSH levels were above 4.5 mIU/L and FT4 levels were below 0.6 ng/dL. Subclinical hyperthyroidism was diagnosed when TSH levels were below 0.45 mIU/L and FT4 levels were between 0.6 and 1.6 ng/dL. Subclinical hypothyroidism was diagnosed when TSH levels were greater than 4.5 mIU/L and FT4 levels were between 0.6 and 1.6 ng/dL (Jain, 2017).

### 2.3 Assessment of cognitive function

In the 2011–2012 NHANES data, the cognitive tests were administered to participants who were 60 years of age or older (CDC, 2012). Assessments are conducted by trained interviewers in mobile testing centers. Three tests were administered: the Alzheimer’s Disease Word List Registry Consortium (CERAD-WL), which assesses immediate and delayed recall of new verbal information (memory subdomain); the Animal Fluency Test, which assess absolute verbal fluency (a component of executive function); and the Digit Symbol Substitution Test (DSST), which assesses processing speed, sustained attention, and working memory. The CERAD test is made up of three tests of continuous learning and a test of delayed recall. Results were therefore presented as three independent trial scores ranging from 0 to 10, total scores for all three trials ranging from 0 to 30, and delayed recall scores ranging from 0 to 10. In practice, Animal Fluency Test scores range from 3 to 39, and the scores of the DSST from 0 to 105, although there is no upper limit.

### 2.4 Covariate data

Covariates included several demographic characteristics: sex, age, race/ethnicity, BMI, PIR, education level, alcohol consumption, and smoking status. Participants were divided into three age groups: 60–69, 70–79, and 80 and older. This study covered Mexicans, non-Hispanic whites, non-Hispanic blacks, and other races (including other Hispanics and multiracial). The World Health Organization (WHO) divides BMI into four categories: underweight (<18.5 kg/m2), normal weight (18.5–25 kg/m2), overweight (25–30 kg/m2), and obese (>30.0 kg/m2). The PIR classifications are < 1 (below the poverty line), 1–1.99, 2–3.99, and 4 (most affluent). The level of education is categorized as less than a high school diploma, a high school diploma/general education diploma, and a college or university-level diploma. Alcohol consumption was divided into four groups: non-drinkers, drinkers of 1 to 5 servings per month, drinkers of 5 to 10 servings per month, or drinkers of more than 10 servings per month. Smoking status was categorized as current, former, or never smoked.

### 2.5 Genetic data on TSH, FT4, hyperthyroidism and hypothyroidism

Genetic data for TSH were obtained from a study authored and published by Williams et al. (2023); the study included 247,107 European subjects and contained a total of 575,241,162 single nucleotide polymorphisms (SNP) (Williams et al., 2023). Genetic data for FT4 were obtained from a study authored and published by Dennis et al. (2021); the study included 26,321 European subjects and contained a total of 6,240,610 single SNP (Dennis et al., 2021). We obtained Genome-Wide Association Studies (GWAS) summary data for hyperthyroidism (1991 patients and 305,175 controls) and hypothyroidism (45,321 patients and 298,847 controls) from the FinnGen study. The FinnGen study is a large-scale genomics initiative that has analyzed over 500,000 Finnish biobank samples and correlated genetic variation with health data to understand disease mechanisms and predispositions. The project is a collaboration between research organizations and biobanks within Finland and international industry partners. The genetic data in our study are summarized in Supplementary Table 1.

### 2.6 Different types of dementia genetic data

The FinnGen study has provided a pool-level GWAS dataset that includes 301,879 participants (16,209 patients and 285,670 controls) with any type of dementia, including AD, FTD, VaD, Senile dementia, simple type, other alcoholic dementia, and delirium superimposed on dementia, etc. Summary statistics for AD were derived from the GWAS database, which included 25,392 patients and 276,086 controls. We obtained GWAS summary data for VaD (2717 patients and 393,024 controls) from the FinnGen study. We obtained GWAS summary data for FTD from the FinnGen study (129 patients and 392,463 controls) and from the IEU database (515 patients and 2,509 controls), respectively. The GWAS data for DLB were obtained from a separate multi-center study that enrolled 2,591 patients and 4,027 controls (Chia et al., 2021)and another study that enrolled 1,180 DLB in APOE e4+ carriers and 657 controls, respectively. The genetic data in our study are summarized in Supplementary Table 1.

### 2.7 Statistical analysis

The R software (4.3.2) was used for all statistical analysis. We used the sample weights for the thyroid profile provided by the NHANES for the weighting. Continuous variables are expressed as the mean or percentage of the population studied. For the survey data in Supplementary Table 2, we performed multiple linear regression analysis, a model used to assess the linear relationship between TSH, FT4 concentrations, and cognitive function. We also used weighted multivariable-adjusted logistic regression to calculate odds ratios (OR) and 95% confidence intervals (95% CI) for cognitive metrics in populations with different thyroid disease groups. Restricted cubic spline (RCS) was used to fit the dose-response relationship between serum TSH and FT4 concentrations and cognitive test scores. In these analyses, we employed three nodes located at the 10th, 50th, and 90th percentiles of the TSH and FT4 distributions. A survey-weighted generalized linear model was applied using the ‘svyglm’ function in R, incorporating restricted cubic splines (RCS) to capture potential non-linear relationships between TSH, FT4, and cognitive test outcomes. The use of restricted cubic splines was selected due to their ability to enforce linearity beyond the boundary knots, thereby enhancing the stability and interpretability of the model at the extremes of the data distribution. All analyses were considered statistically significant at *p* < 0.05, using the NHANES complex multistage sampling design.

In our study, we performed univariate two-sample MR analysis to determine the causal relationship between genetically predicted hyperthyroidism, hypothyroidism, TSH and FT4 concentration and dementia (any dementia, AD, VD, FTD and DLB) using the TwoSampleMR R package (Emdin et al., 2017). An overview of the design of the study is shown in Figure 1B. MR analyses must meet three requirements to investigate the causal effect of exposure on outcomes: (1) genetic variants should be associated with hyperthyroidism, hypothyroidism, TSH and FT4 concentration; (2) they should not be associated with confounders; and (3) they should only affect dementia mediated by hyperthyroidism, hypothyroidism, TSH and FT4 concentration. The following procedure was used to select genetic variants prioritized to meet the three instrumental assumptions of MR analysis (Burgess et al., 2017b). First, SNPs associated with hyperthyroidism, hypothyroidism, TSH and FT4 concentration at the genome-wide significance level (*p* < 5e–8) were extracted as instrumental variables (IV). Second, SNPs with linkage disequilibrium (r2 threshold < 0.001 within a 10000kb window) were eliminated, and the remaining SNPs were extracted from the outcome datasets. Third, we calculated the F-statistic for each SNP to quantify the strength of the association and excluded SNPs that were weak (*F*-statistic < 10).

We then primarily used the Inverse Variance Weighting (IVW) method, which has the strongest analytic power but no detectable horizontal pleiotropy by itself, to assess the relationship between hyperthyroidism, hypothyroidism, TSH and FT4 concentration and dementia (any dementia, AD, VD, FTD, and DLB). The Wald ratio method was used when there was only one instrumental SNP available for analysis. We also use MR-Egger regression and weighted median (WME) for further analysis to test the robustness of the results. MR-Egger regression and WME can detect and adjust for horizontal pleiotropy, which is the simultaneous effect of a genetic variant (or instrumental variable) on multiple traits or outcomes through multiple independent pathways. However, both MR-Egger and WME are not as statistically valid as IVW. To assess potential heterogeneity and pleiotropy, sensitivity analysis is essential. In addition, heterogeneity between causal estimates of different genetic variants was assessed using Cochran’s Q test (Burgess et al., 2017a). Furthermore, leave-one-out (LOO) cross-validation was used for sensitivity analysis to verify the reliability of the IVW results, which can identify potentially unusual instrumental variables and assess the robustness of results by eliminating each instrumental variable individually and examining the change in causal estimates after each elimination. In addition, we employed Mendelian Randomization Pleiotropy RESidual Sum and Outlier (MR-PRESSO) to further investigate the potential horizontal pleiotropic effect indicated by the leave-one-out (LOO) analysis. This test was conducted for each SNP to determine the presence of significant horizontal pleiotropic bias, with corresponding p-values provided. Additionally, the aggregate horizontal pleiotropic effect of all SNPs was evaluated, and the presence of significant pleiotropic bias was ascertained by calculating the global p-value.

## 3 Results

### 3.1 General characteristics of NHANES

In the 2011–2012 NHANES cycle, 409 individuals aged 60 years or older with complete cognitive function scores and data on TSH and FT4 concentrations and information on important covariates including gender, age, race/ethnicity, smoking status, alcohol use, and education were selected for this study. The participants in this study were relatively evenly distributed by age and gender; the majority (50%) of the population in this study were between 60 and 69 years of age, while 203 (49.6%) were female and 206 (50.4%) were male, as shown in Table 1. In this study, most of the participants were non-Hispanic white, accounting for nearly 82% of the population. The number of underweight people is very minimal, with about one-third having a normal body mass index, more than one-third being overweight, and about one-third being obese. Most participants consume very little alcohol and either have never smoked or are former smokers. One-third of the population has a university degree, and about one-third has a degree higher than a university degree. FT4 and TSH levels were at normal levels in most people, while the median FT4 level in the population was 0.85 ng/dL and the median TSH level was 1.77 mIU/L. Across the three recall trials (with a maximum score of 30), the median CERAD total score was 19. The median scores for the CERAD delayed recall, Animal Fluency Test, and DSST were 6, 17, and 52, respectively. The average cognitive scores are typical for cognitively healthy individuals (Christensen et al., 2020).

**TABLE 1:** NHANES 2011–2012 participant baseline characteristics and clinical data.

Characteristic	Overall	Female	Male
**N (Unweighted)**	409 (100%)	203 (49.6%)	206 (50.4%)
**Age.group**			
60–69 years	210 (50%)	113 (55%)	97 (44%)
70–79 years	115 (30%)	44 (22%)	71 (40%)
80+ years	84 (20%)	46 (24%)	38 (16%)
**Sex**			
Female	203 (49.6%)		
Male	206 (50.4%)		
**Race**			
Non-Hispanic White	183 (82%)	96 (82%)	87 (81%)
Non-Hispanic Black	113 (7.7%)	54 (8.2%)	59 (7.1%)
Other Hispanic	46 (4.1%)	23 (3.8%)	23 (4.3%)
Other/multiracial	42 (4.3%)	16 (3.4%)	26 (5.4%)
Mexican American	25 (2.3%)	14 (2.4%)	11 (2.1%)
**BMI.group**			
Underweight (<18.5)	4 (2.2%)	4 (4.1%)	0 (0%)
Normal (18.5 to<25)	119 (29%)	55 (29%)	64 (29%)
Overweight (25 to<30)	139 (37%)	62 (35%)	77 (40%)
Obese (30 or greater)	140 (31%)	79 (32%)	61 (31%)
**Alq.group**			
1–5 drinks/month	193 (42%)	84 (40%)	109 (44%)
5–10 drinks/month	20 (7.2%)	7 (6.2%)	13 (8.5%)
10+ drinks/month	70 (24%)	20 (16%)	50 (33%)
Non-drinker	122 (27%)	89 (37%)	33 (15%)
**Smoke.group**			
Current smoker	54 (10%)	16 (7.1%)	38 (14%)
Former smoker	168 (45%)	59 (35%)	109 (57%)
Never smoker	187 (45%)	128 (58%)	59 (29%)
**Education.attainment**			
Less Than 9th Grade	58 (8.0%)	24 (6.7%)	34 (9.5%)
9–11th Grade	69 (12%)	35 (10%)	34 (15%)
High School Grad/GED	79 (18%)	46 (21%)	33 (14%)
Some College or AA degree	117 (32%)	66 (39%)	51 (24%)
College Graduate or above	86 (29%)	32 (23%)	54 (38%)
**FT4 Group(ng/dL)**			
**<0.60**	5 (2.0%)	2 (1.1%)	3 (3.0%)
**0.60–1.60**	400 (97%)	198 (96%)	202 (97%)
**> 1.60**	4 (1.4%)	3 (2.4%)	1 (0.2%)
**TSH Group(mIU/L)**			
**< 0.45**	12 (4.5%)	6 (5.6%)	6 (3.2%)
**0.45–4.5**	380 (91%)	192 (92%)	188 (89%)
**> 4.5**	17 (4.7%)	5 (2.4%)	12 (7.4%)
**Age**	68.9 (64.0, 74.0)	68.0 (64.0, 74.2)	70.0 (64.2, 74.0)
**FT4(ng/dL)**	0.85 (0.76, 0.94)	0.86 (0.76, 0.95)	0.84 (0.77, 0.90)
**TSH(mIU/L)**	1.77 (1.20, 2.53)	1.62 (1.15, 2.47)	1.98 (1.40, 2.58)
**PIR**	3.04 (1.62, 4.76)	2.75 (1.44, 4.67)	3.14 (1.80, 4.71)
**BMI**	27.5 (24.1, 31.0)	26.7 (24.1, 32.0)	27.7 (24.6, 30.8)
**CERAD1**	5.00 (4.00, 6.00)	5.00 (4.00, 6.00)	4.00 (3.62, 6.00)
**CERAD2**	7.00 (6.00, 8.00)	7.00 (6.00, 8.00)	6.00 (5.00, 7.00)
**CERAD3**	7.20 (7.00, 9.00)	8.00 (7.00, 9.00)	7.00 (6.00, 8.00)
**CERAD.total**	19.0 (16.0, 22.0)	20.7 (17.0, 23.0)	18.0 (15.0, 20.0)
**CERAD.delay.recall**	6.00 (4.00, 8.00)	7.00 (5.00, 8.00)	5.00 (4.00, 7.00)
**Animal.Fluency**	17.0 (14.0, 21.0)	17.0 (13.7, 20.0)	18.0 (14.2, 22.0)
**DSST**	52 (40, 66)	54 (41, 70)	49 (40, 60)

### 3.2 Relationship between serum TSH and FT4 concentrations and cognitive function

As shown in Table 2, the CERAD1 score was significantly different among the groups with TSH concentration < 0.45 mIU/L, 0.45–4.5 mIU/L, and > 4.5mIU/L (P = 0.028), with median values of 6, 5, 4, respectively. The CERAD.delay.recall score was also significantly different among these three groups (*P* < 0.001), with median values of 7, 6, 6, respectively. Moreover, the CERAD3 score significantly differed among the groups with FT4 concentration < 0.6 ng/dL, 0.6–1.6 ng/dL and > 1.6 ng/dL (*P* = 0.002), with median values of 7.32, 7.22, 5.86, respectively. The Animal Fluency Test score was also significantly different among these three groups stratified by FT4 concentration (*P* < 0.001) with median values of 17.7, 17, and 10.6, respectively. The overall differences between the three groups may reveal broad trends or patterns that may not be apparent in a two-by-two comparison due to statistical power, adjusted significance levels, or the complexity of the data. In fact, Table 3 only suggests that CERAD1 was higher in the group with TSH concentration less than 0.45 mIU/L than in the normal group (0.45–4.5 mIU/L), which is partially consistent with the results in Table 2.

**TABLE 2:** Multivariable-adjusted logistic regression to compare cognitive function among groups with different serum TSH and FT4 concentrations.

Characteristic	TSH(mIU/L)			*P*	FT4(ng/dL)			*P*
**Group**	**<0.45**	**0.45–4.5**	**>4.5**		**<0.60**	**0.60–1.60**	**>1.60**	
**N(Unweighted)**	12	380	17		4	400	4	
CERAD1	6.00 (5.00, 6.96)	5.00 (4.00, 6.00)	4.00 (4.00, 5.00)	0.028*	4.64 (2.90, 5.75)	5.00 (4.00, 6.00)	4.30 (3.00, 4.93)	0.5
CERAD2	7.00 (6.00, 7.00)	7.00 (5.00, 8.00)	6.14 (6.00, 7.53)	>0.9	6.64 (5.49, 7.75)	7.00 (6.00, 8.00)	6.30 (4.00, 6.93)	0.4
CERAD3	8.00 (6.48, 8.00)	7.51 (7.00, 9.00)	7.00 (6.68, 7.17)	0.2	7.32 (6.49, 7.88)	7.22 (7.00, 9.00)	5.86 (5.00, 7.46)	0.002*
CERAD.total	20.2 (18.0, 23.0)	19.0 (16.0, 22.0)	17.1 (16.8, 19.5)	0.2	18.6 (16.0, 21.4)	19.0 (16.0, 22.0)	18.3 (12.0, 18.9)	0.068
CERAD.delay.recall	7.00 (7.00, 7.00)	6.00 (4.00, 8.00)	6.00 (4.52, 6.57)	<0.001*	6.51 (4.05, 7.00)	6.00 (4.00, 8.00)	4.29 (3.00, 6.00)	0.052
Animal Fluency: Score Total	20.0 (16.0, 22.2)	17.0 (14.0, 21.1)	15.2 (13.6, 16.4)	0.10	17.7 (12.1, 24.5)	17.0 (14.0, 21.0)	10.6 (10.0, 12.4)	<0.001*
DSST	65 (42, 85)	52 (40, 66)	42 (40, 54)	0.3	59 (32, 69)	52 (40, 65)	38 (27, 49)	0.6

The table describes the median and 25th and 75th percentiles of cognitive function test scores for different serum TSH and FT4 concentrations. Multivariable-adjusted logistic regression to compare cognitive function among groups with different serum TSH and FT4 concentrations. Fully adjusted models are adjusted for survey cycle, age, sex, alcohol intake, smoking, PIR, and education.

*P < 0.05. This bold values suggest *P* < 0.05, which is statistically significant.

**TABLE 3:** Adjusted odds ratios (95% Confidence Intervals) for cognitive function test scores across TSH and FT4 groups.

	CERAD1	CERAD2	CERAD3	CERAD.total	CERAD.delay. recall	Animal.Fluency	DSST
**TSH(mIU/L)**							
**<0.45 vs. 0.45–4.5**	2.32 (1.06, 5.10)*	0.56 (0.24, 1.32)	0.70 (0.37, 1.31)	0.92 (0.11, 7.51)	0.96 (0.47, 1.97)	0.22 (0.01, 3.48)	116 (0.00, 4, 408, 641)
**>4.5 vs. 0.45–4.5**	1.01 (0.44, 2.32)	1.30 (0.58,2.94)	1.22 (0.55, 2.72)	1.61 (0.16,16.2)	1.74 (0.69, 4.37)	0.06 (0.00, 2.16)	0.82 (0.02, 27.5)
**>4.5 vs<0.45**	0.43 (0.16, 1.18)	2.33 (0.67, 8.07)	1.75 (0.72, 4.23)	1.75 (0.09, 33.1)	1.81 (0.64, 5.07)	0.28 (0.00, 27.9)	0.01 (0.00, 224)
**FT4 (ng/dL)**							
**<0.60 vs 0.60–1.60**	1.63 (0.47, 5.62)	1.57 (0.43,5.66)	0.96 (0.35, 2.59)	2.44 (0.08,70.5)	2.11 (0.40, 11.0)	6.76 (0.05, 967)	455 (0.00,71,361,943)
**>1.60 vs 0.60–1.60**	1.40 (0.86, 2.29)	0.74 (0.28,1.94)	0.74 (0.51, 1.06)	0.76 (0.19, 3.11)	0.93 (0.53, 1.64)	0.13 (0.01, 1.71)	3.39 (0.00, 3,393,235)
**>1.60 vs<0.60**	0.86 (0.21, 3.48)	0.47 (0.09, 2.37)	0.77 (0.25, 2.42)	0.31 (0.01, 14.5)	0.44 (0.07, 2.74)	0.02 (0.00, 12.7)	0.01 (0.00, 1,505,660)

Adjusted Odds Ratios (95% Confidence Intervals) for cognitive function test scores across TSH and FT4 groups. Fully adjusted models are adjusted for survey cycle, age, sex, alcohol intake, smoking, PIR, and education.

*P < 0.05. This bold values suggest *P* < 0.05, which is statistically significant.

### 3.3 Relationship between thyroid disorders and cognitive function

As shown in Table 4, weighted logistic regression results from the NHANES database showed no significant association between thyroid disease and cognitive function.

**TABLE 4:** The effects of various thyroid diseases on cognitive function.

Characteristic	Group	exp(Beta)	SE	t	*p*
CERAD1	Normal	ref	ref	ref	ref
	Hyperthyroidism	1.798	0.892	2.015	0.293
	Hypothyroidism	0.591	0.311	1.904	0.308
	Subhyperthyroidism	0.757	0.508	1.491	0.376
	Subhypothyroidism	−0.062	0.556	−0.112	0.929
CERAD2	Normal	ref	ref	ref	ref
	Hyperthyroidism	−0.100	0.477	−0.210	0.868
	Hypothyroidism	0.933	0.410	2.275	0.264
	Subhyperthyroidism	−0.624	0.543	1.149	0.456
	Subhypothyroidism	0.178	0.558	0.319	0.803
CERAD3	Normal	ref	ref	ref	ref
	Hyperthyroidism	1.006	0.306	3.283	0.188
	Hypothyroidism	1.042	0.285	3.650	0.170
	Subhyperthyroidism	−0.482	0.392	−1.229	0.435
	Subhypothyroidism	0.095	0.566	0.169	0.894
CERAD.total	Normal	ref	ref	ref	ref
	Hyperthyroidism	2.711	1.577	1.718	0.336
	Hypothyroidism	2.552	0.887	2.876	0.213
	Subhyperthyroidism	−0.337	1.337	−0.252	0.843
	Subhypothyroidism	0.214	1.584	0.135	0.914
CERAD.delay.recall	Normal	ref	ref	ref	ref
	Hyperthyroidism	1.073	0.540	6.152	0.297
	Hypothyroidism	3.564	0.501	7.110	0.089
	Subhyperthyroidism	−0.139	0.452	−0.309	0.809
	Subhypothyroidism	0.161	0.648	0.248	0.845
Animal.Fluency	Normal	ref	ref	ref	ref
	Hyperthyroidism	−0.196	1.001	−0.196	0.877
	Hypothyroidism	−0.748	1.392	−0.538	0.686
	Subhyperthyroidism	−1.650	1.815	−0.909	0.530
	Subhypothyroidism	−3.066	2.329	−1.316	0.414
DSST	Normal	ref	ref	ref	ref
	Hyperthyroidism	−1.746	3.062	−0.570	0.670
	Hypothyroidism	−8.042	2.686	−2.994	0.205
	Subhyperthyroidism	5.348	6.717	0.796	0.572
	Subhypothyroidism	0.809	2.566	0.315	0.806

Fully adjusted models are adjusted for survey cycle, age, sex, alcohol intake, smoking, PIR, and education. The exp (Beta) coefficient indicates the strength and direction of the linear relationship between different types of thyroid diseases and cognitive function. SE (Standard Error) is a measure of the uncertainty in estimating the beta coefficient. It represents the standard deviation between the estimated beta value in the sample and its overall mean. The *t* is a statistic used to test whether the beta coefficient is significantly different from 0.

### 3.4 Dose-response relationship analysis

**FIGURE 2:**
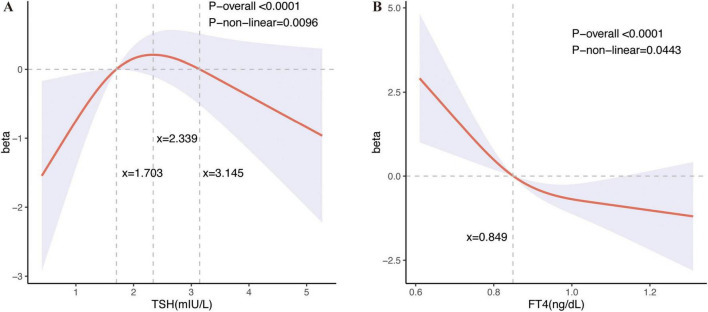
Restricted cubic spline plots between serum TSH and FT4 levels and the results of the Animal Fluency Test. Restricted cubic spline (RCS) regression characterized dose-response relationships between serum thyroid stimulating hormone (TSH) concentrations and FT4 concentrations and Animal Fluency Test. Fully adjusted models are adjusted for survey cycle, age, sex, alcohol intake, smoking, PIR, and education. The beta coefficient delineates the varying effects of serum TSH and FT4 concentrations on the Animal Fluency Test across different intervals. The horizontal gray line, positioned at zero on the Y-axis, represents a beta value of 0. **(A)** Dose-response relationships between serum TSH concentrations and Animal Fluency Test: The three vertical gray lines in the graph denotes specific key values for TSH concentrations (x = 1.703, x = 2.339, x = 3.145 mIU/L), P-non-linear = 0.0096. Serum TSH concentrations in the range of 1.703 to 3.145 mIU/L showed a positive correlation with performance on the Animal Fluency Test, with the most pronounced positive correlation observed at a TSH level of approximately 2.339 mIU/L, whereas TSH concentrations outside this range showed a negative correlation with the Animal Fluency Test. **(B)** Dose-response relationships between serum FT4 concentrations and Animal Fluency Tes: The vertical gray line in the graph indicates a key value for FT4 concentration (x = 0.849 ng/dL), *P*-non-linear = 0.0443. Animal Fluency Test scores were positively correlated with FT4 on the left side of the serum FT4 concentration inflection point (0.849 ng/L), whereas on the right side, they were positively correlated.

There was no linear relationship between serum TSH and FT4 concentration and cognitive function, as shown in Supplementary Table 2. Therefore, restricted cubic spline (RCS) was used to analyze the relationship between serum TSH concentrations and FT4 concentrations and Animal Fluency Test scores. Based on this relationship, the nonlinear model explained the relationship better than the linear model after adjusting for all covariates (TSH: *P*-non-linear = 0.0096; FT4: *P*-non-linear = 0.0443) (Figure 2). The serum TSH concentrations within the range of 1.703 to 3.145 mIU/L exhibit a positive correlation with Animal Fluency Test, whereas concentrations outside this range are negatively correlated with Animal Fluency Test. The serum FT4 concentrations exhibited a positive correlation with Animal Fluency Test to the left of the serum FT4 concentration inflection point (0.849 ng/L), whereas to the right of this inflection point, correlation was negative. We additionally conducted RCS analyses of TSH and FT4 concentrations in relation to CERAD1, CERAD2, CERAD3, CERAD total, CERAD delayed recall, and DSST scores. The results showed a non-linear but statistically insignificant relationship between serum TSH and CERAD1, but serum FT4 concentration showed a significant non-linear relationship with CERAD1. Furthermore, both serum TSH and FT4 concentrations were nonlinearly related to CERAD2, with significant results for TSH. In contrast, the nonlinear relationship between serum FT4 concentration and CERAD2 was only weakly significant. However, the nonlinear associations between serum TSH and FT4 concentrations and the scores of CERAD3, CERAD total, CERAD delayed recall, and DSST were not statistically significant (Supplementary Figure 1).

### 3.5 MR analysis of the causal effect of genetically determined thyroid disease and serum TSH concentrations on the risk of developing dementia

**FIGURE 3:**
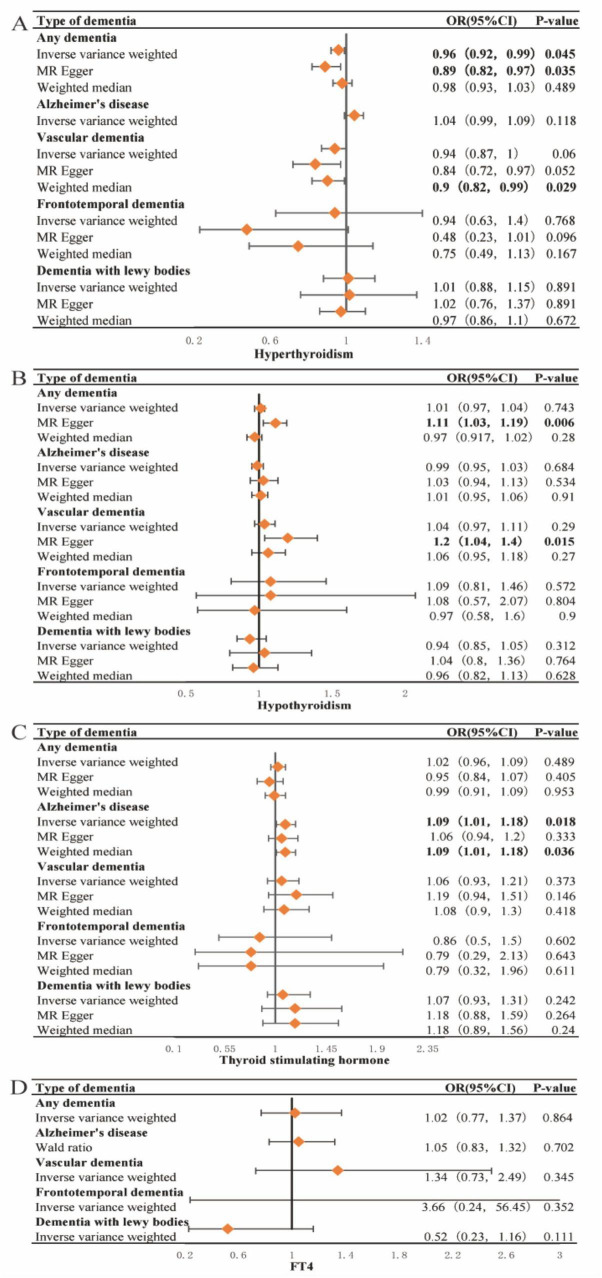
Forest plots of Mendelian Randomization analysis investigating the effects of hyperthyroidism, hypothyroidism, TSH and FT4 concentrations on each type of dementia. Forest plots of Mendelian Randomization (MR) in our study, including Inverse Variance Weighting (IVW), MR Egger, and Weighted median. **(A)** Forest plots of MR analysis of the correlation of hyperthyroidism with each type of dementia; It is showed that hyperthyroidism may reduce the risk of dementia and vascular dementia. **(B)** Forest plots of MR analysis of the correlation of hypothyroidism with each type of dementia; It is showed that hypothyroidism may increase the risk of dementia and vascular dementia. **(C)** Forest plots of a MR analysis of the correlation of thyroid stimulating hormone (TSH) concentrations with each type of dementia; It is showed that elevated concentrations of TSH may increase the risk of AD. **(D)** Forest plots of a MR analysis of the correlation of FT4 concentrations with each type of dementia; there was no significant causal association between the FT4 concentrations and the risk of developing each type of dementia. *P*-value < 0.05 is statistically significant. OR, odds ratio; CI, confidence interval.

The results of the primary IVW analysis indicated that genetic predisposition to hyperthyroidism was associated with a 4% reduction in odds of dementia [OR = 0.96, 95% CI: 0.92–0.99, *P* = 0.045] (Figure 3A), and that genetic predisposition to TSH concentrations was associated with a 9% increase in odds of AD [OR = 1.09, 95% CI: 1.01–1.18, *P* = 0.018] (Figure 3C). For the association between hyperthyroidism and dementia, the findings of MR-Egger [OR = 0.89, 95% CI: 0.82–0.97, *P* = 0.035] (Figure 3A) were consistent with the IVW method. The results of MR-Egger suggest that genetic predisposition to hypothyroidism was associated with an 11% increase in odds of dementia [OR = 1.11, 95% CI: 1.03–1.19, *P* = 0.006] and a 20% increase in odds of VaD [OR = 1.2, 95% CI: 1.04–1.4, *P* = 0.015] (Figure 3B). The results of WME suggest that genetic predisposition to hyperthyroidism was associated with a 10% reduction in odds of VaD [OR = 0.9, 95% CI: 0.82–0.99, *P* = 0.029] (Figure 3A). And for the association between TSH concentrations and AD, the findings of WME [OR = 1.09, 95% CI: 1.01–1.18, *P* = 0.036] (Figure 3C) was consistent with the IVW method. No cross-sectional pleiotropy (p-intercept > 0.05) was found for most of the selected instruments in the sensitivity analysis (Supplementary Tables 3–7). However, MR analysis suggested that there was no significant causal relationship between FT4 concentration and the risk of developing each type of dementia. Heterogeneity in tools selected for hypothyroidism and VaD and for TSH and dementia and AD. The forest plot generated by the LOO analysis shows the effect value (beta value) after excluding SNPs one by one (Supplementary Figures 2–4). When we exclude SNPs one by one, the black dots represent the effect estimate (i.e., beta value) for the remaining SNPs after excluding the corresponding SNPs, and the black line indicates the confidence interval for this estimate. In the association of hyperthyroidism with dementia and VaD, the effect estimate changed significantly and became nonsignificant after removal of SNP rs1794511. This may indeed indicate horizontal pleiotropy or the potential for this SNP to serve as a null instrumental variable. The MR-Egger intercept p-values were 0.026 and 0.043, respectively, suggesting slight evidence of pleiotropy. However, the primary results of the MR-Egger analysis did not show strong horizontal pleiotropy. In addition, further analysis using MR-PRESSO yielded a global test result of *p* = 0.483, indicating no significant overall horizontal pleiotropy in the causal relationship between hyperthyroidism and VaD. A global test with a *p*-value of 0.05 for the causal relationship between hyperthyroidism and dementia suggested borderline significance, indicating a potential slight pleiotropy; however, no significant aberrant SNPs were identified (Supplementary Table 7). Despite the absence of significant pleiotropy following the exclusion of individual SNPs (e.g., rs1794511), certain effect sizes exhibited some variation. Nevertheless, the combined results of the LOO and MR-Egger analyses consistently demonstrated that the overall causal effect remained robust.

## 4 Discussion

To our knowledge, this is the first study based on data from a large-scale observational study and MR analysis based on large-scale genetic data to comprehensively examine the association of thyroid disorders, TSH and FT4 concentrations with cognitive function and the risk of developing dementia. We investigated the relationship of serum TSH and FT4 concentrations with cognitive function. The results of this study showed statistically significant differences in both CERAD1 (*P* = 0.028) and CERAD.delay.recall (*P* < 0.001) among groups with serum TSH concentrations < 0.45 mIU/L, 0.45–4.5 mIU/L and > 4.5 mIU/L. Meanwhile, the results showed that there was a statistically significant difference in both CERAD3 (*P* = 0.002) and Animal Fluency Test scores (*P* < 0.001) among serum FT4 concentrations < 0.6 ng/dL, 0.6–1.6 ng/dL, and > 1.6 ng/dL groups. And the results suggest that the low serum TSH concentrations and serum FT4 concentrations groups had higher cognitive function scores. Surprisingly, our analysis identified a potential nonlinear association between serum TSH and FT4 concentrations with the CERAD2 scores and Animal Fluency Test scores. Additionally, a comparable association was observed between serum FT4 concentrations and CERAD1 scores, which partially elucidates the elevated CERAD1 scores in the FT4 intermediate group (0.6–1.6 ng/dL), as indicated by Table 2. Simultaneously, MR analysis results also suggest that genetic predisposition to TSH concentrations may increase the risk of AD. In addition, this large observational study also examined the relationship between various thyroid conditions and cognitive function and the results were not statistically significant. However, MR analysis indicated that genetic predisposition to hyperthyroidism may be associated with a reduced risk of dementia and VaD, whereas genetic predisposition to hypothyroidism may be linked to an increased risk of these conditions.

Previous studies have shown that both subclinical hyperthyroidism and hypothyroidism due to abnormal serum TSH concentrations increase the risk of AD and VaD (Tan et al., 2008). Our results based on MR analysis suggest that genetic predisposition to hypothyroidism and increased levels of TSH concentrations are associated with increased risk of VaD and AD, respectively, which is in line with the study of Tan et al. In addition, our findings indicate a non-linear association between serum TSH concentrations and cognitive function. Specifically, serum TSH concentrations within the range of 1.703 to 3.145 mIU/L exhibit a positive correlation with Animal Fluency Test, whereas concentrations outside this range are negatively correlated with Animal Fluency Test. This pattern aligns with previous research suggesting that abnormal TSH concentrations may increase the risk of AD and VaD, although genetically predicted TSH concentrations that are increased within the normal range are associated with a reduced risk of AD (Li et al., 2021; Marouli et al., 2021). Several studies have identified T4 concentrations as an important cognitive and dementia risk factor in older adults (Prinz et al., 1999; Hogervorst et al., 2008). Previous studies have reported an association between high FT4 concentrations and accelerated cognitive decline and dementia progression (Hogervorst et al., 2008). The results of the present study are also consistent with previous findings, but interestingly, the relationship between serum FT4 concentrations and cognitive impairment may be nonlinear, with the negative correlation with cognitive impairment gradually flattening at higher serum FT4 concentrations. However, the MR analysis results suggest that there was no significant causal association between the FT4 concentrations and the risk of developing each type of dementia. The inconsistency between the results of MR analysis and cross-sectional studies on the relationship between FT4 concentrations and cognitive function may be due to the small number of SNPs used as instrumental variables within the MR analyses. For the diagnosis and treatment of dementia associated with thyroid dysfunction and other risk factors, serum TSH concentrations and FT4 concentrations may serve as potential research and clinical targets.

Thyroid disease is diagnosed on the basis of serum TSH and FT4 concentrations, and thus thyroid disease is affected by the complexity of the relationship between serum TSH and FT4 concentrations. The association between serum TSH concentrations and CREAD2 scores exhibited a plateau pattern, whereas the relationship between serum FT4 concentrations and CREAD2 scores demonstrated an essentially L-shaped configuration. Conversely, the correlation between serum TSH concentrations and Animal Fluency Test scores followed an inverted U-shaped, while the association between serum FT4 concentrations and Animal Fluency Test scores remained fundamentally L-shaped. This discrepancy can be ascribed to the varying cognitive domains represented by different scores, suggesting that the impacts of TSH and FT4 may differ across these distinct cognitive domains. Thus, in terms of the non-linear relationship between serum TSH and FT4 concentrations on cognitive function alone, thyroid disease is protective against dementia when serum TSH concentrations reduces the risk of dementia more than serum FT4 concentrations increases the risk of dementia, or when serum FT4 concentrations reduces the risk of dementia more than serum TSH concentrations increases the risk of dementia, but the effects of thyroid disease on cognitive function may be mediated by other, more complex influencing factors, and serum TSH and FT4 concentrations may have an interactive effect. This may partially elucidate the inconsistencies and ongoing controversies observed in current studies examining the relationship between thyroid disease and cognitive decline. Despite numerous studies indicating either an association (Kalmijn et al., 2000; van Osch et al., 2004; Quinlan et al., 2020) or a lack thereof (Yoshimasu et al., 1991; de Jong et al., 2006; Annerbo et al., 2009; Brenowitz et al., 2018) between hyperthyroidism/hypothyroidism, the precise relationship between Alzheimer’s disease (AD) and thyroid function remains inconclusive. Recent systematic reviews have demonstrated that the effects of hypothyroidism on AD can be investigated through various mechanisms, including diminished neuromodulation, altered gene expression, impaired autophagy, increased oxidative stress, and brain metabolic dysfunction. Importantly, these factors do not operate in isolation; instead, they interact synergistically to contribute to the onset and progression of AD (Li and Liu, 2024). Observational results of the study suggest no association between different types of thyroid disorders and cognitive dysfunction, but some of the results of MR analysis were statistically significant.

Both the IVW and MR-Egger methods of MR analysis indicate that genetic predisposition to hyperthyroidism is associated with a decreased risk of developing dementia. This finding implies the presence of a robust and consistent causal relationship, or alternatively, that pleiotropy is insufficient to significantly impact this association. MR analysis results using solely WME prompts, revealing that genetic predisposition to hyperthyroidism is associated with a reduced risk of developing VaD. The lack of significant findings using IVW and MR-Egger methods could be attributed to potential pleiotropy within the instrumental variables, resulting in inconsistent effects of these variables on the exposure-outcome relationship. However, the WME method, owing to its robustness and reduced sensitivity to such inconsistencies, may yield more reliable estimates. The MR analysis found that the protective effect of hyperthyroidism against dementia and VaD may be related to the fact that thyroid hormones can increase systemic metabolism, stimulate angiogenesis, and improve vascular endothelial function. The results of the study are less well established in the literature, and more studies need to be conducted to confirm the results. In the MR analysis, only the MR-Egger method indicated that genetic predisposition to hypothyroidism increases the risk of dementia and VaD, potentially reflecting the influence of multivalence or variability in the direction of the effects of instrumental variables. This observation aligns with prior results from an extensive case-control study conducted by Wieland et al., which identified an 81% elevated risk of dementia among individuals aged 65 years or older with a history of hypothyroidism (Wieland et al., 2022). In the interim, the outcomes derived from the IVW and WME methods in the MR analysis indicate that genetic predisposition to elevated TSH concentrations may elevate the risk of Alzheimer’s Disease (AD). This observation suggests that the instrumental variables demonstrate a robust causal relationship between TSH concentrations and AD, characterized by an absence of significant evidence of pleiotropy and a consistent direction of effect. However, neither genetic predisposition to hypothyroidism nor genetic predisposition to hyperthyroidism is associated with AD. This difference may be due to differences in the pathological basis of the different types of dementia, the effects of thyroid function on the cardiovascular and metabolic systems, the sensitivity and statistical power of the data, the heterogeneity of the effects of the instrumental variables, and differences in specific biological mechanisms. Furthermore, although the observed odds ratio (OR) reached statistical significance, its proximity to 1 suggests a potentially limited benefit. This observation could be attributed to the large sample size, the small effect size, random error or noise within the data, and the presence of potential confounders or biases. Consequently, additional clinical trials are warranted to corroborate our findings.

There may be several reasons for the discrepancy between MR analysis and cross-sectional results. First, although the heterogeneity of the instruments selected for hypothyroidism and VaD as well as for TSH concentrations and dementia and AD does not fully explain the inconsistency between the results of the MR analysis and the results of the cross-sectional study. The heterogeneity of the tools selected for hypothyroidism and VaD may be related to the pleiotropy of MR analysis. The heterogeneity of tools selected for TSH concentrations and AD may be related to factors such as different gene-environment interactions or differences in the effects of tool variables. The heterogeneity may influence the reliability of the findings regarding the elevated risk of VaD associated with genetic predisposition to hypothyroidism, as well as the increased risk of AD linked to genetic predisposition to TSH concentrations. However, it does not compromise the reliability of the other results. Second, the assumption of MR analysis is usually that the relationship between genetic predisposition to TSH concentrations, and each type of dementia is linear and stable over time, but the results of the present cross-sectional study suggest that the relationship between serum TSH concentrations and cognitive function is nonlinear, which explains the inconsistency between the results of the MR analysis and those of the cross-sectional study. Third, the inconsistency between the results of MR analysis and the cross-sectional study may be due more to the small sample size of the cross-sectional study. Therefore, a larger sample size is needed to confirm the results of this study.

Some strengths of this study include the large sample size, the inclusion of several types of dementia, and the use of SNPs as genetic instrumental variables. Importantly, we were able to assess the causal effect of TSH concentrations, hyperthyroidism, and hypothyroidism on the risk of developing dementia using MR analysis. However, this study has some limitations. First, we conducted the MR analysis using data from people of European descent, whereas the cross-sectional study used data from multiracial Americans. Studies of individuals of the same race are needed to eliminate potential confounders of population heterogeneity. Second, a larger population is needed for a RCT because the cross-sectional studies have relatively few complete data on both cognitive and thyroid function. Third, the incidence of FTD and DLB may be low, so the number of cases is relatively small. All the data in the database have been used in this paper, and the results suggest that there is a certain trend between thyroid disease and the risk of FTD, but there is no consistent statistical significance, so large-scale clinical RCT trials are needed to verify the results. Fourth, due to database limitations, the current study did not include real-time patient samples to explain the relationship between dementia and hormones. Fifth, Our study is limited by the absence of a mechanistic component involving wet-lab experiments on blood samples from dementia patients, relying predominantly on statistical analysis.

This study provides evidence of a nonlinear relationship between serum TSH and FT4 concentrations and cognitive function, with hyperthyroidism decreasing the risk of dementia and VaD, hypothyroidism increasing the risk of dementia and VaD, and elevated TSH concentrations increasing the risk of AD. To validate the findings of this study, it is imperative to conduct extended and larger-scale RCT. Additionally, there is a critical need for more comprehensive basic research across various levels, including molecular, cellular, and animal models, to progressively elucidate the pathomechanisms underlying the association between thyroid dysfunction and dementia. Such research endeavors will facilitate the identification of a comprehensive and compelling network of mechanisms, ultimately contributing to the development of more effective clinical treatment strategies. Furthermore, prioritizing early detection, diagnosis, and treatment through the assessment of thyroid function in individuals at high risk for developing dementia is of paramount importance. This strategy has the potential to significantly contribute to the prevention and deceleration of dementia progression.

## Data Availability

The datasets presented in this study can be found in online repositories. The names of the repository/repositories and accession number(s) can be found in the article/Supplementary material.
