# Supplementary material for: Hyperthyroidism, hypothyroidism, thyroid stimulating hormone, and dementia risk: results from the NHANES 2011–2012 and Mendelian randomization analysis
Source: Front Aging Neurosci. 2024 Oct 23;16:1456525. doi: 10.3389/fnagi.2024.1456525 (PMC11538144; doi:10.3389/fnagi.2024.1456525)

Supplementary Material

# Supplementary Data

**Table S1**. The GWAS details of dementia in the Mendelian randomization

| **Trait** | **Population** | **Cases** | **Controls** | **Sample size** | **PubMed ID or web source** |
| --- | --- | --- | --- | --- | --- |
| Hyperthyroidism | European | 1,991 | 305,175 | 307,166 | www.finngen.fi/en |
| Hypothyroidism | European | 45,321 | 298,847 | 359,822 | www.finngen.fi/en |
| Thyroid stimulating hormone levels | European | NA | NA | 247,107 | https://www.ebi.ac.uk/gwas/studies/GCST90296333 |
| FT4 | European | NA | NA | 26,231 | https://www.ebi.ac.uk/gwas/studies/GCST90012662 |
| Any dementia | European | 16,209 | 285,670 | 301,879 | www.finngen.fi/en |
| Alzheimer's disease | European | 25,392 | 276,086 | 301,478 | https://www.ebi.ac.uk/gwas/studies/GCST90027158 |
| Vascular dementia | European | 2,717 | 393,024 | 395,741 | www.finngen.fi/en |
| Frontotemporal dementia | European | 129 | 392,463 | 392,592 | www.finngen.fi/en |
|  | European | 515 | 2,509 | 3,024 | https://gwas.mrcieu.ac.uk/datasets/ieu-b-43/ |
| Dementia with Lewy bodies | European | 2,591 | 4,027 | 6,618 | https://www.ebi.ac.uk/gwas/studies/GCST90001390 |
|  | European | 1,180 | 657 | 1,837 | https://www.ebi.ac.uk/gwas/studies/GCST90093378 |

# Supplementary Figures and Tables

## **Table S2**. Multiple linear regression analysis of TSH, FT4 concentrations and cognitive function

|  | **TSH** | | | **FT4** | | |
| --- | --- | --- | --- | --- | --- | --- |
| **Characteristic** | **Beta** | **95% CI** | **P** | **Beta** | **95% CI** | **P** |
| CERAD1 | 0.0041 | -0.2117, 0.2200 | 0.9601 | -0.1583 | -1.5275, 1.2108 | 0.7642 |
| CERAD2 | 0.1142 | -0.0967, 0.3250 | 0.2072 | -0.7813 | -2.3367, 0.7744 | 0.2357 |
| CERAD3 | 0.0784 | -0.0640, 0.2207 | 0.2012 | -0.4647 | -1.6388, 0.7095 | 0.3336 |
| CERAD.total | 0.1979 | -0.2994, 0.6952 | 0.3312 | -1.3934 | -5.1515, 2.3646 | 0.3614 |
| CERAD.delay.recall | 0.0693 | -0.1525, 0.2911 | 0.4348 | 0.2273 | -1.67891, 2.1336 | 0.7572 |
| Animal.Fluency | -0.1404 | -0.6478, 0.3671 | 0.4853 | -4.5551 | -10.3249, 1.2147 | 0.0935 |
| DSST | 0.1716 | -1.2379, 1.5812 | 0.7523 | 6.046 | -10.7371, 22.8291 | 0.3738 |

**Table S3.** Heterogeneity and pleiotropy tests for the Hyperthyroidism with dementia.

| **Outcome** | **SNPs** | **Cochrane’s Q test** | | | **MR-Egger intercept test** | |
| --- | --- | --- | --- | --- | --- | --- |
|  |  | **Q-value** | **P _Q_** |  | **Intercept** | **P _intercept_** |
| Any dementia | 9 | 16.724 | 0.151 |  | 0.026 | 0.118 |
| Alzheimer's disease | 2 | 0.073 | 0.787 |  | NA | NA |
| Vascular dementia | 9 | 3.693 | 0.814 |  | 0.043 | 0.141 |
| Frontotemporal dementia | 9 | 8.721 | 0.273 |  | 0.257 | 0.087 |
| Dementia with Lewy bodies | 7 | 10.863 | 0.054 |  | -0.005 | 0.927 |

**Table S4.** Heterogeneity and pleiotropy tests for the Hypothyroidism with dementia

| Outcome | **SNPs** | Cochrane’s Q test | | | MR-Egger intercept test | |
| --- | --- | --- | --- | --- | --- | --- |
|  |  | Q-value | P _Q_ |  | Intercept | P _intercept_ |
| Any dementia | 149 | 172.822 | 0.071 |  | -0.008 | 0.004 |
| Alzheimer's disease | 53 | 150.086 | 0.414 |  | -0.003 | 0.376 |
| Vascular dementia | 149 | 18.42 | 0.010 |  | -0.0135 | 0.0281 |
| Frontotemporal dementia | 149 | 122.545 | 0.930 |  | 0.257 | 0.087 |
| Dementia with Lewy bodies | 127 | 143.408 | 0.137 |  | -0.008 | 0.435 |

**Table S5**. Heterogeneity and pleiotropy tests for the serum thyroid stimulating hormone concentrations with dementia

| **Outcome** | **SNPs** | **Cochrane’s Q test** | | | **MR-Egger intercept test** | |
| --- | --- | --- | --- | --- | --- | --- |
|  |  | **Q-value** | **P _Q_** |  | **Intercept** | **P _intercept_** |
| Any dementia | 143 | 201.024 | 0.0007 |  | 0.004 | 0.145 |
| Alzheimer's disease | 43 | 76.859 | 0.0006 |  | 0.002 | 0.540 |
| Vascular dementia | 143 | 154.905 | 0.191 |  | -0.006 | 0.200 |
| Frontotemporal dementia | 143 | 140.793 | 0.489 |  | 0.005 | 0.834 |
| Dementia with Lewy bodies | 127 | 131.438 | 0.329 |  | -0.004 | 0.584 |

**Table S6**. Heterogeneity and pleiotropy tests for the serum FT4 concentrations with dementia

| **Outcome** | **SNPs** | **Cochrane’s Q test** | | | **MR-Egger intercept test** | |
| --- | --- | --- | --- | --- | --- | --- |
|  |  | **Q-value** | **P _Q_** |  | **Intercept** | **P _intercept_** |
| Any dementia | 2 | 1.115 | 0.291 |  | NA | NA |
| Vascular dementia | 2 | 0.622 | 0.430 |  | NA | NA |
| Frontotemporal dementia | 2 | 0.112 | 0.737 |  | NA | NA |
| Dementia with Lewy bodies | 2 | 0.086 | 0.769 |  | NA | NA |

**Table S7**. MR-PRESSO Analysis of the Causal Relationship Between Hyperthyroidism and Dementia/VaD

| **Outcome** | **Analysis Type** | **MR Analysis Causal Estimate (beta)** | **Standard Deviation (SD)** | **T-Statistic** | **Raw P-Value** | **Global Test P-Value** | **Global Test RSSobs** |
| --- | --- | --- | --- | --- | --- | --- | --- |
| Any dementia | beta.exposure | -0.044 | 0.022 | -2.005 | 0.080 | 0.05 | 25.117 |
|  | beta.exposure Outlier-corrected | NA | NA | NA | NA |  |  |
| Vascular dementia | beta.exposure | -0.066 | 0.031 | -2.095 | 0.069 | 0.483 | 10.222 |
|  | beta.exposure Outlier-corrected | NA | NA | NA | NA |  |  |

## Supplementary Figures

**Figure S1**. Restricted cubic spline plots between serum TSH and FT4 levels and the results of CERAD1, CERAD2, CERAD3, CERAD total, CERAD delayed recall, and DSST.


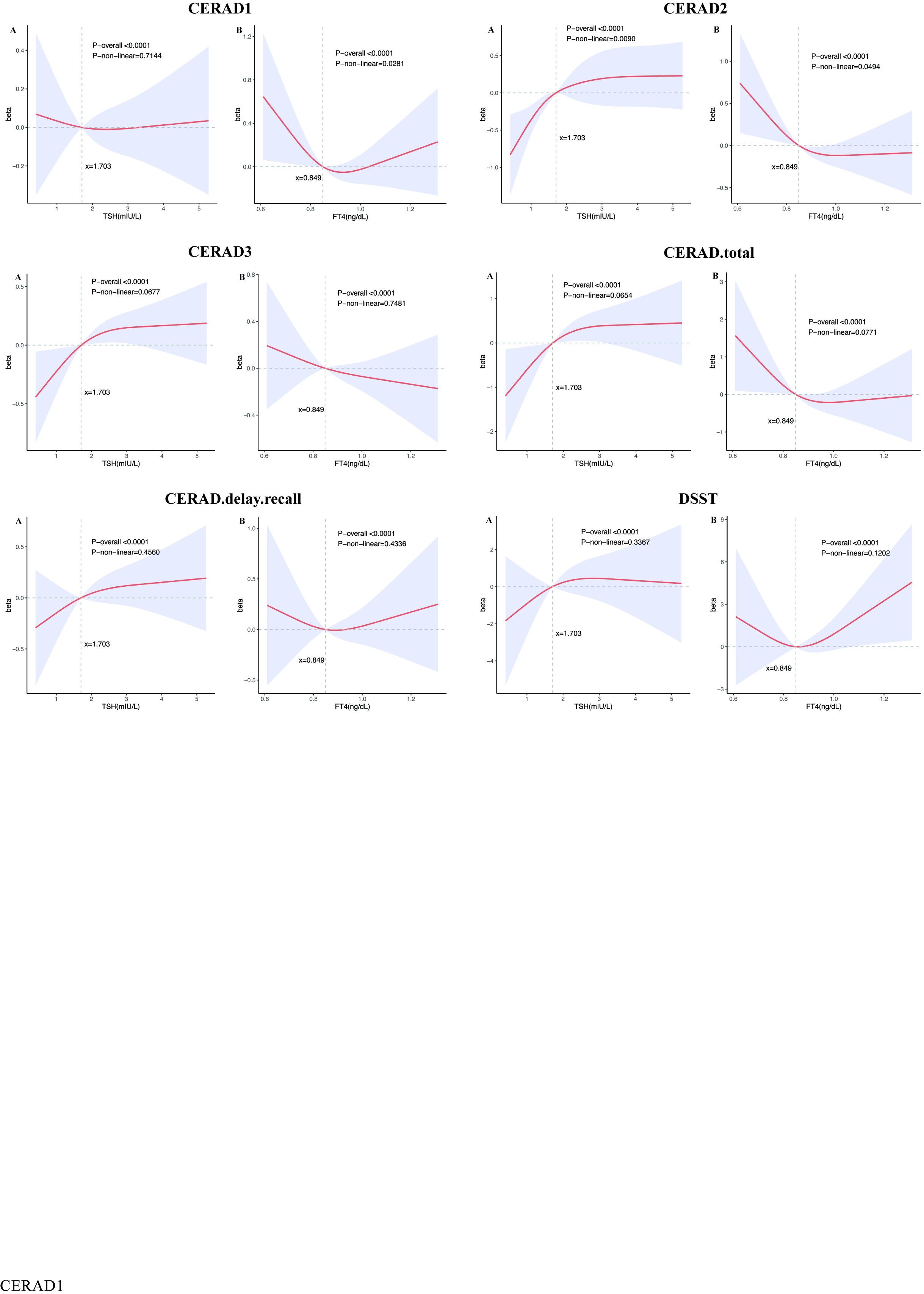


Restricted cubic spline(RCS) regression characterized dose-response relationships between serum thyroid stimulating hormone (TSH) concentrations and FT4 concentrations and the results of CERAD1, CERAD2, CERAD3, CERAD total, CERAD delayed recall, and DSST. Fully adjusted models are adjusted for survey cycle, age, sex, alcohol intake, smoking, PIR, and education.

**Figure S2**. Sensitivity testing of instrumental variables for hyperthyroidism and any dementia, VaD, FTD, and DLB using the leave-one-out method


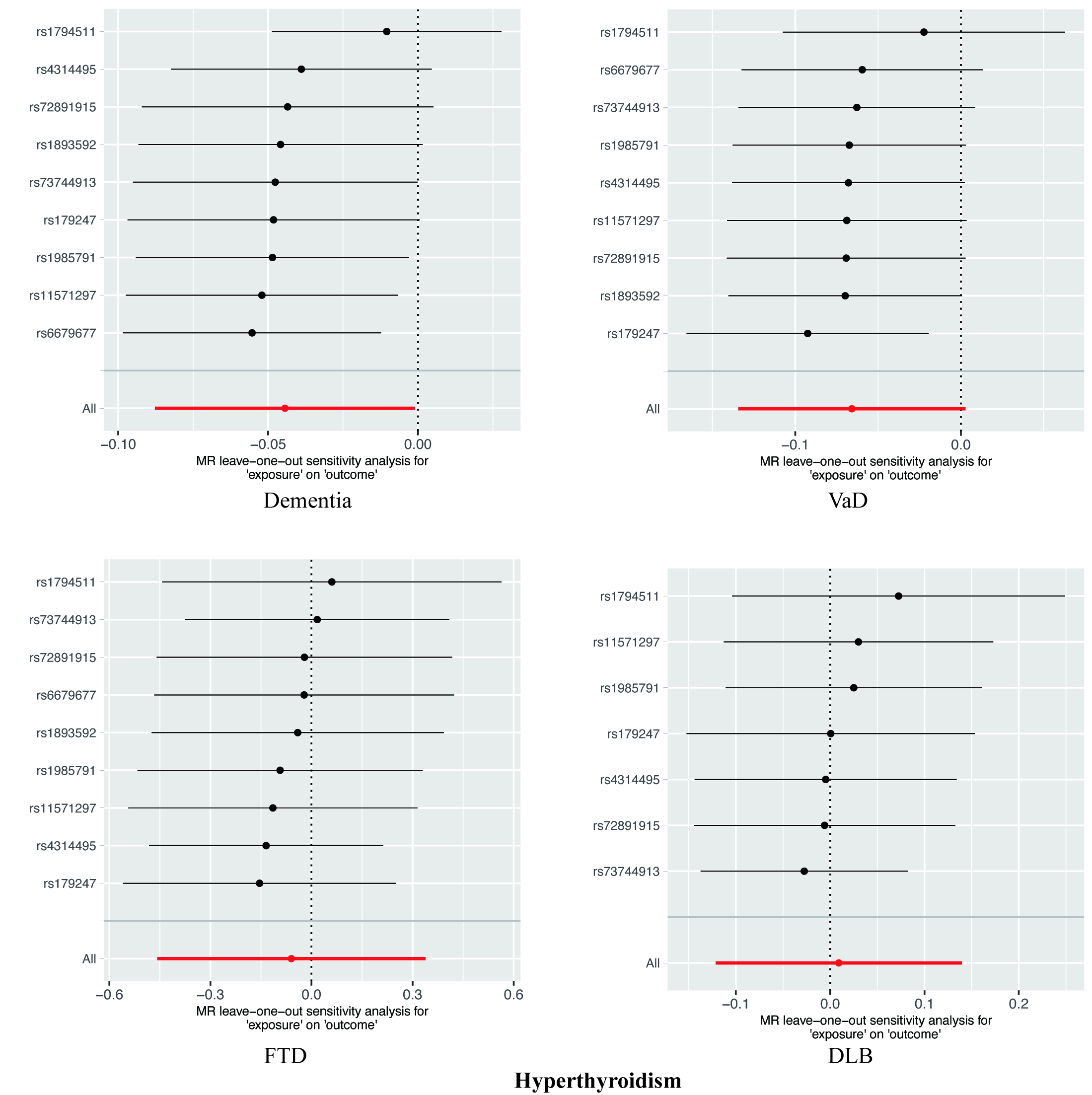


**Figure S3**. Sensitivity testing of instrumental variables for hypothyroidism and any dementia, VaD, FTD, and DLB using the leave-one-out method


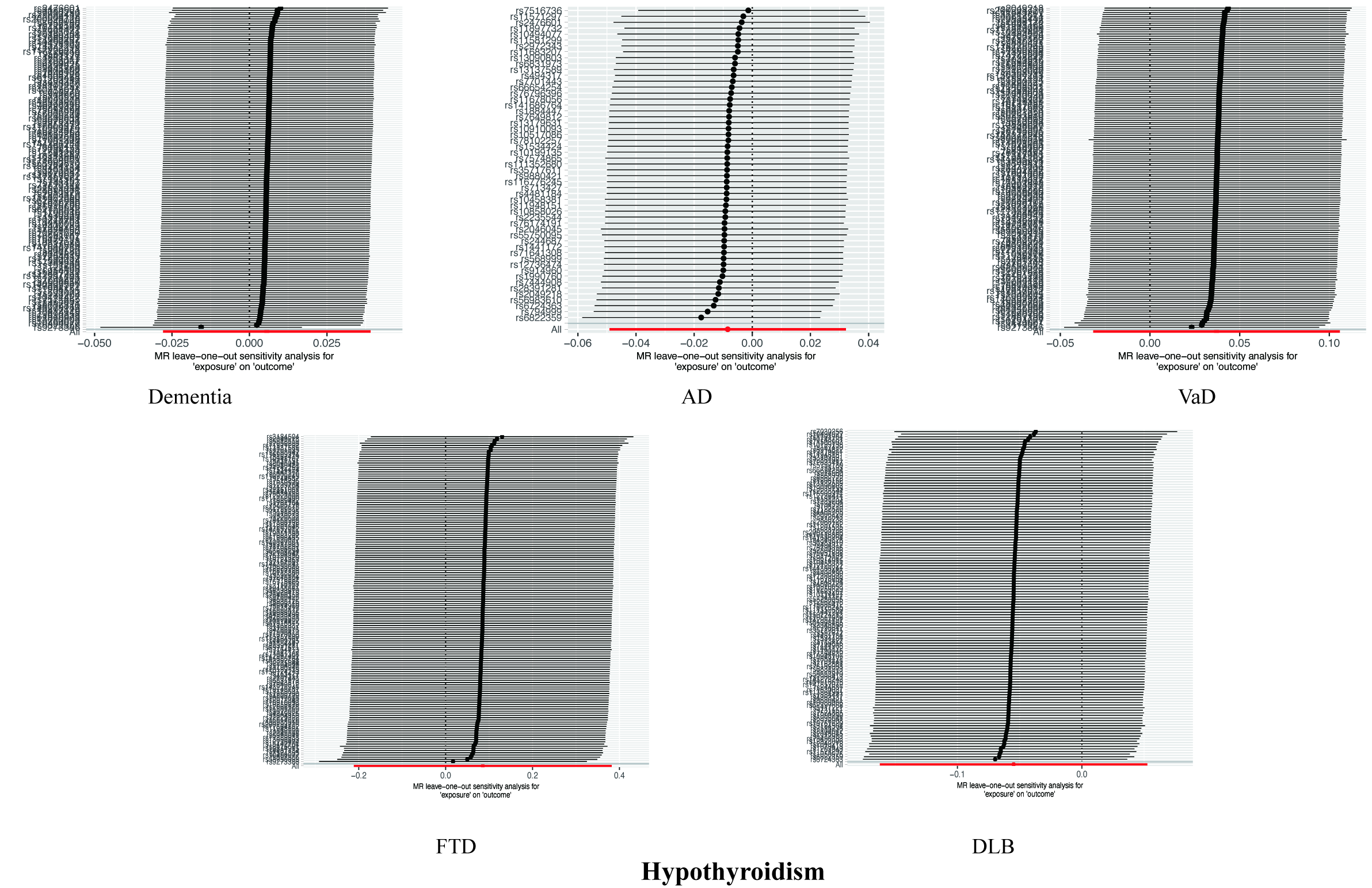


**Figure S4**. Sensitivity testing of instrumental variables for concentrations of thyroid stimulating hormone and any dementia, VaD, FTD, and DLB using the leave-one-out method


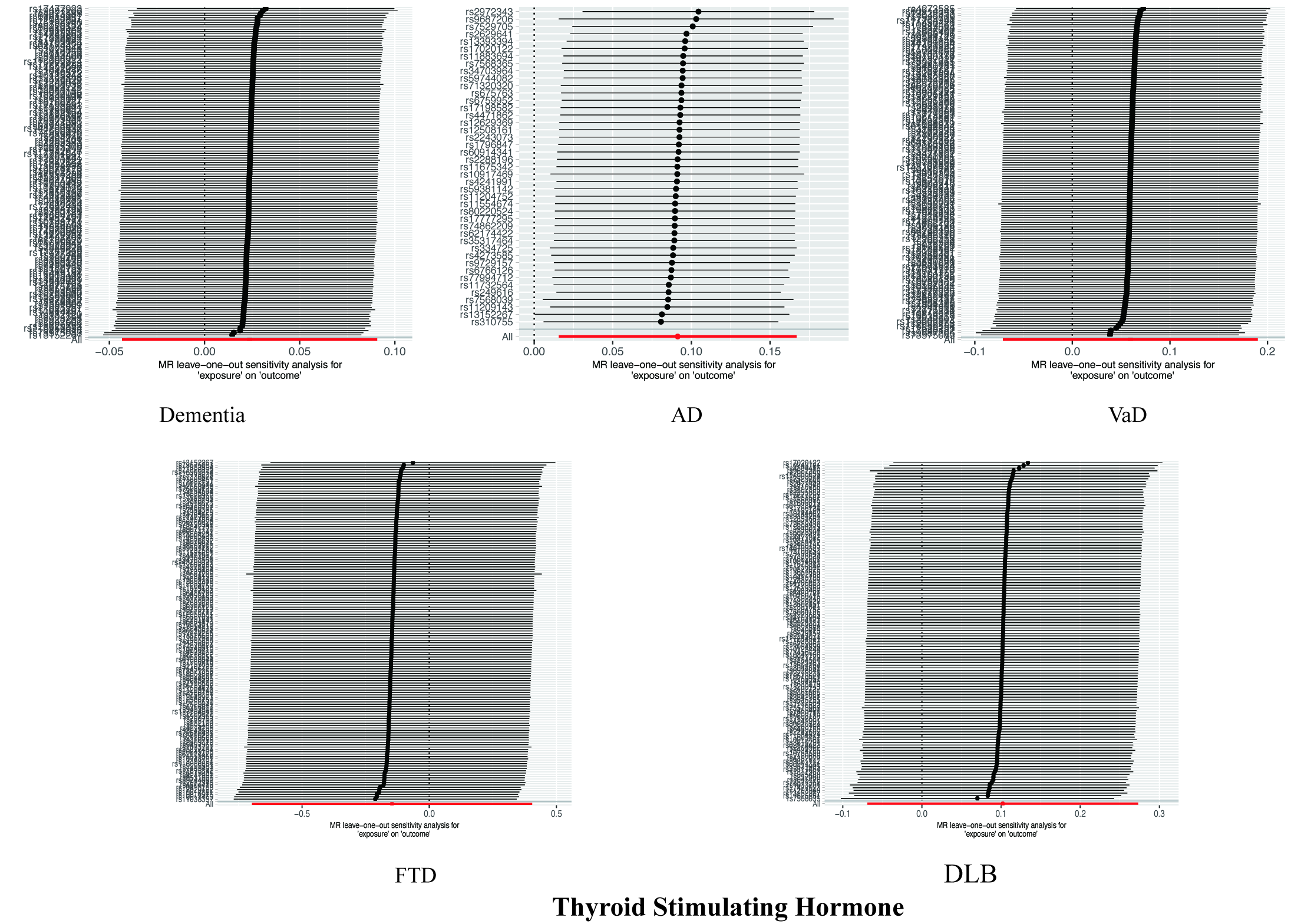

Supplement: Supplementary file 1 [file Table_1.DOCX]
